# Transcriptional Activity of Heparan Sulfate Biosynthetic Machinery is Specifically Impaired in Benign Prostate Hyperplasia and Prostate Cancer

**DOI:** 10.3389/fonc.2014.00079

**Published:** 2014-04-15

**Authors:** Anastasia V. Suhovskih, Alexandra Y. Tsidulko, Olesya S. Kutsenko, Anna V. Kovner, Svetlana V. Aidagulova, Ingemar Ernberg, Elvira V. Grigorieva

**Affiliations:** ^1^Institute of Molecular Biology and Biophysics SD RAMS, Novosibirsk, Russia; ^2^Novosibirsk State University, Novosibirsk, Russia; ^3^Research Center of Clinical and Experimental Medicine SD RAMS, Novosibirsk, Russia; ^4^Novosibirsk State Medical University, Novosibirsk, Russia; ^5^MTC, Karolinska Institute, Stockholm, Sweden

**Keywords:** heparan sulfate, proteoglycan, biosynthesis, expression, transcriptional pattern, prostate cancer

## Abstract

Heparan sulfates (HSs) are key components of mammalian cells surface and extracellular matrix. Structure and composition of HS, generated by HS-biosynthetic system through non-template-driven process, are significantly altered in cancer tissues. The aim of this study was to investigate the involvement of HS-metabolic machinery in prostate carcinogenesis. Transcriptional patterns of HS-metabolic enzymes (EXT1, EXT2, NDST1, NDST2, GLCE, 3OST1/HS3ST1, SULF1, SULF2, HPSE) were determined in normal, benign, and cancer human prostate tissues and cell lines (PNT2, LNCaP, PC3, DU145). Stability of the HS-metabolic system patterns under the pressure of external or internal stimuli was studied. Overall impairment of transcriptional activity of HS-metabolic machinery was detected in benign prostate hyperplasia, while both significant decrease in the transcriptional activity and changes in the expression patterns of HS metabolism-involved genes were observed in prostate tumors. Prostate cancer cell lines possessed specific transcriptional patterns of HS metabolism-involved genes; however, expression activity of the system was similar to that of normal prostate PNT2 cells. HS-metabolic system was able to dynamically react to different external or internal stimuli in a cell type-dependent manner. LNCaP cells were sensitive to the external stimuli (5-aza-deoxycytidin or Trichostatin A treatments; co-cultivation with human fibroblasts), whereas PC3 cells almost did not respond to the treatments. Ectopic GLCE over-expression resulted in transcriptional activation of HS-biosynthetic machinery in both cell lines, suggesting an existence of a self-regulating mechanism for the coordinated transcription of HS metabolism-involved genes. Taken together, these findings demonstrate impairment of HS-metabolic system in prostate tumors *in vivo* but not in prostate cancer cells *in vitro*, and suggest that as a potential microenvironmental biomarker for prostate cancer diagnostics and treatment.

## Introduction

Heparan sulfate proteoglycans (HSPGs) play an important role in cell–cell and cell–matrix communication and signaling, being an essential part of the cell microenvironment (1, 2). A functional specificity of interactions of heparan sulfate (HS) with multiple cell surface and extracellular ligands is related to the fine structure of the HS polysaccharide chains, generated by the system of HS-synthesizing and modifying enzymes (3, 4). Because of non-template nature of HS biosynthesis, functional capabilities of the HS-metabolic machinery is vital for proper HS synthesis and functions, and understanding of HS biosynthesis regulation is important for the understanding of the HSPGs functions (5–7).

During carcinogenesis, significant changes in HS structure, composition, and functional activity occur (8–11), and distortion of the HS-biosynthetic machinery could be a first candidate for the changes. Many different enzymes (multiple glycosyltransferases, sulfotransferases, and an epimerase) participate in HS biosynthesis and catalyze the assembly and modification of HS chains (12, 13). All the enzymes work together and each participant provides the preferred substrate for the next reaction in the pathway, forming highly informative HS chains. A physical complex of the enzymes, committed to the assembly of HS, was designated as “GAGosome” (14, 15).

Recently, a direct involvement of individual HS-metabolic enzymes in carcinogenesis was demonstrated, although for some enzymes the obtained data are still controversial. Potential tumor-suppressor function is shown for EXT1, NDST4, GLCE, and SULF1 genes and changes of their expression were detected in different human tumors: EXT1 is epigenetically inactivated in leukemia and non-melanoma skin human primary tumors and cell lines (16) and mutationally inactivated in solid osteochondromas (17), but EXT1 expression is associated with bad prognosis in multiple myeloma cells (18); NDST4 is a novel candidate tumor-suppressor gene, and the loss of its function might be involved in colorectal cancer progression (19); GLCE is down-regulated in both breast primary tumors and cell lines (20, 21) and lung cancer cell lines (22), although GLCE up-regulation correlates with aggressive disease in prostate cancer (23, 24); SULF1 is epigenetically silenced in ovarian cancer cell lines and primary tumors (25); in contrast to the tumor-suppressor effect of SULF1, SULF2 expression promotes hepatocellular carcinoma cell growth *in vitro* and *in vivo* (26); SULF2 is up-regulated in NSCLC and other cancers and implicated as a driver of carcinogenesis in NSCLC, pancreatic cancer, and hepatocellular carcinoma (27); enhanced heparanase-1 expression correlates with poor prognosis in most of the cancers in which it has been examined, and over-expression and knockout studies clearly implicate the heparanase as a master regulator of cancer progression and metastasis (28, 29). All the results support an involvement of HS-metabolic enzymes in carcinogenesis and show complex changes of their expression levels in tumors.

Till date, only few studies were performed to reveal transcriptional patterns for HS-metabolic machinery in normal and cancer tissues. Complex characterization of expression profiles of 100 genes, involved in synthesis of glycosaminoglycan (GAG) chains, was done for normal and malignant human plasma cells using Affymetrix microarray. It was shown that expression of nine of these genes – *EXT2*, *CHSY3*, *CSGALNACT1*, *HS3ST2*, *HS2ST1*, *CHST11*, *CSGALNACT2*, *HPSE*, *SULF2* – are significantly different between normal and malignant plasma cells (17). Expression analysis of a set of 419 functionally relevant genes involved in synthesis, degradation, and binding of N-linked and O-linked glycans, Lewis antigens, GAGs (chondroitin, heparin, and keratan sulfate in addition to hyaluronan) and glycosphingolipids revealed that mRNA levels for many of them differ significantly between normal and malignant breast tissue (30). Transcriptomic and histochemical approaches were used to analyze the expression of the enzymes involved in HS and chondroitin sulfate (CS) biosynthesis and editing, as well as the proteoglycan core proteins, in metastatic and non-metastatic breast adenocarcinomas. No significant change in transcription was detected in approximately 70% of analyzed genes, however, 13 genes demonstrated changes in both tumor types (syndecan-1, glypican-3, perlecan, NDST4, 3-*O*-sulfotransferase) and 5 genes showed changes only in non-metastatic tumors (sulfatases), expression of heparanase-2 but not heparanase-1 was significantly deregulated (31). Expression of nine HS-modifying enzymes (HPSE, 2OST1, 3OST1, 3OST3B, 6OST1, NDST1 and 2, Sulf1 and 2) was heterogeneously changed in human fibrogenic diseases and hepatocellular carcinoma, with intensified expression of several HS-modifying enzymes (NDST1 and -2, 3OST1, Sulf1) in pathological tissues (32). A study on the expression of HS-synthesizing enzymes in the development of *Nematostella vectensis* shows that the expression levels of *EXT1*, *EXT2*, *NDSTa*, *NDSTb*, *C5 epimerase*, and *2OSTa* are rather uniform at all stages, but *6OST*, *3OST*, and *Sulf* display elevated expression levels during blastula and gastrula stages (33). The expression of HS sulfotransferases (3-*O*-sulfotransferase-1 [3OST1], -2, and -4; 6OST1, -2, and -3; and *N*-deacetylase/*N*-sulfotransferase-1 [NDST1], -2, and -3) is spatiotemporally regulated in the development of the nervous system, suggesting spatiotemporal changes of the HS structure and various biological activities in the developing mouse brain (34). At the moment, it is almost all publications in this field.

The results suggest a hypothesis that not only expression levels of individual enzymes but also their ratio and functional balance are of importance in successfully working HS-metabolic system. These individual changes seem to disbalance HS metabolism and create cancer-specific transcriptional patterns of HS-metabolic system in cancer tissues.

In this study, we investigated the common expression patterns of key HS metabolism-involved genes in normal, benign, and cancer human prostate tissues and cell lines. Stability of the system upon different external or internal stimuli was studied as a possible molecular mechanism of dynamic HS structure/composition changes in prostate cancer.

## Materials and Methods

### Patients and tissue samples

All tissue samples were obtained from primary tumors during radical surgery at the Central Municipal Hospital N1, Novosibirsk, Russia. Tissues were “snap-frozen” in liquid nitrogen and stored at –70°C. Regions were manually dissected from the frozen blocks to provide a consistent tumor cell content of more than 70% for analysis. The majority of prostate cancer patients were at the II–III stage of malignancy progression according the TNM staging system. PSA was 0.1–8.5 ng/ml for BPH and 1.8–50 ng/ml for adenocarcinoma patients, Gleason scores were 2–9. Normal prostate tissue samples were obtained from normal prostates surgically resected by medical indications during non-prostate surgery. All patients provided written informed consent and the study protocol was approved by the Local Ethics Committee in accordance with the Helsinki Declaration of 1975.

### Analysis of HS metabolism-involved genes expression using RT-PCR

Total RNA was extracted from the cells using the PureLink Total RNA Purification System (Invitrogen, Carlsbad, CA, USA) according to the manufacturer’s instructions. cDNA was synthesized from 1 to 2 μg of total RNA using a First Strand cDNA Synthesis kit (Fermentas, Hanover, MD, USA) and 1/10th of the product was subjected to PCR analysis.

The following conditions were used for RT-PCR: 95°C for 10 min, 95°C for 15 s, 55–64°C for 15 s, and 72°C for 1 min, with a final elongation step at 72°C for 10 min using a Tercik PCR machine (DNA-Technology, Russia). The total reaction volume was 20 μl. The amplified products were separated on 1.2% agarose gels. The gels were scanned using the “DNA Analyzer” system (Moscow, Russia) and genes expression levels were estimated from the intensity of the amplified DNA fragment normalized against the intensity of *GAPDH*.

The PCR primers and conditions are listed in Table 1.

**Table 1:** **Primer sequences and PCR conditions**.

Gene	Sequences	DNA product, bp	PCR conditions	
			Annealing *T*, °C	Cycles
EXT1	F 5’-TTGGGTCCTTCAGATTCCTG-3’	329	59	33
	R 5’-TCCTCCAGGATGTTTGTTCC-3’	
EXT2	F 5’-AAGCACCAGGTCTTCGATTACC-3’	296	55	33
	R 5’-GAAGTACGCTTCCCAGAACCA-3’	
NDST1	F 5’-CACACAGAACGAACTACGC-3’	513	64	33
	R 5’-CCCGTTGATGATCTTGTCC-3’	
NDST2	F 5’-GCCTCCAGTTCCACCTC-3’	575	64	33
	R 5’-CGACGAAGAACTGGTCC-3’	
GLCE	F 5’-CTACACAATGGGGACCTCAAGGC-3’	665	59	34
	R 5’-GCCACCTTTCTCATCCTGGTTCC-3’	
3-OST1	F 5’-CGGGTCTCAGTGGGTGCCTG-3’	382	64	33
	R 5’-ATCCTGGAGGGTCCCCGCTT-3’	
SULF1	F 5’-CTCACAGTCCGGCAGAGCAC-3’	371	59	33
	R 5’-CACGGCGTTGCTGCTATCTGC-3’	
SULF2	F 5’-GAGGCAGATTCACGTCGTTTCCA-3’	302	59	33
	R 5’-ATCTGGTGCTTCTTTTGGGATGCGGGAG-3’	
HPSE	F 5’-TTCGATCCCAAGAAGGAATC-3’	720	59	33
	R 5’-ATAAAGCCAGCTGCAAAGGT-3’	
GAPDH	F 5’-GGGCGCCTGGTCACCAG-3’	350	59	22
	R 5’-AACATGGGGGCATCAGCAGAG-3’			

### Cell lines, cell culture, and 5-aza-dC/TSA treatment

The PNT2 normal human prostate epithelial cell line was obtained from the European Collection of Cell Cultures (ECACC, Salisbury, UK). The human immortalized fibroblasts, LNCaP, PC3, and DU145 cell lines were obtained from MTC (Karolinska Institute, Sweden). The cell lines were maintained in RPMI medium supplemented with l-glutamine, 100 units/ml penicillin, 100 μg/ml streptomycin, and 10% FBS at 37°C in a humidified 5% CO_2_ incubator. Treatment with deoxyazacytidine (5-aza-dC 2 μg/ml) or Trichostatin A (TSA, 250 ng/ml) was performed by incubating the cells with the drugs for 72 or 24 h, respectively. Cells were harvested using trypsin/EDTA.

### Transfection and selection of stable cell clones

To obtain stable LNCaP and PC3 cell clones expressing *GLCE*, epi-pCEP4 plasmid was used (21). Transfection and selection of stable cell clones were performed as described earlier (24). Briefly, LNCaP and PC3 cells were transfected with epi-pCEP4 or pCEP4 plasmid DNA (0.5 μg DNA per well) in 12-well plates using Lipofectamine and Plus Reagent (Invitrogen, USA) according to the manufacturer’s protocol. Transiently transfected epi-LNCaP, pCEP4-LNCaP and epi-PC3, pCEP4-PC3 cells were cultured for 2–3 weeks in RPMI medium containing Hygromycin (400 μg/ml) to select stable clones.

### Analysis of *GLCE* expression using multiplex RT-PCR

Multiplex RT-PCR analysis of *GLCE* expression was performed as previously described (24). Total RNA was extracted using the PureLink Total RNA Purification System (Invitrogen), cDNA was synthesized using a First Strand cDNA Synthesis kit (Fermentas). The PCR primers used were: GLCE-F, 5’-AAGGGAGACGAGAGGGGAACGAA-3’; GLCE-R, 5’-GCCACCTTTCTCATCCTGGTTC-3’; GAPDH-F, 5’-GGGCGCCTGGTCACAA-3’; GAPDH-R, 5’-AACATGGGGGCAT CAGCAGA-3’.

### Immunostaining

For immunohistochemistry, 3- to 3.5-μm sections of formalin-fixed, paraffin-embedded tissue sections were deparaffinized and antigen was retrieved by treatment with unmasking solution at 95–98°C for 20 min. Immunostaining was performed using biotin-free polyvalent DAB system (Spring Bioscience, USA) according to the manufacturer’s instructions. Briefly, endogenous peroxidase was blocked by incubation with peroxidase inhibitor (30 min at 37°C), unspecific staining was blocked with 5% Fetal Bovine Serum in PBS (20 min at 37°C), and the primary anti-HS mouse monoclonal antibody (Abnova, USA) (1:200) and secondary anti-mouse HRP (1:2000) were used for immunostaining (1 h at 37°C). Staining patterns were visualized by incubation with DAB (15 min at 37°C), counterstained with hematoxylin–eosin, and observed by light microscopy (Axiostar Plus, Carl Zeiss).

### Statistical analysis

Statistical analyses were performed using a computer program ORIGIN Pro 8.1; a value of *p* < 0.05 was considered to indicate a statistically significant difference. Data are expressed as the means ± SEM. Differences between the means of three different groups of patients were analyzed using ANOVA.

## Results

### Heparan sulfate expression in normal and pathological human prostate tissues

**Figure 1:**
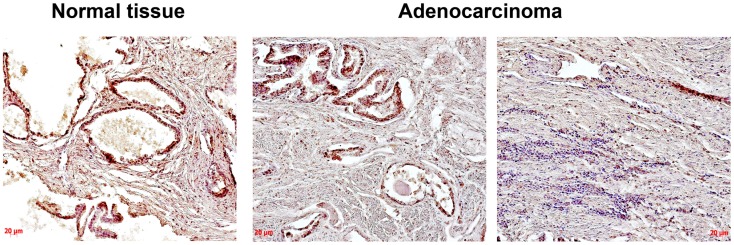
**Immunocytochemical analysis of heparan sulfate expression in normal human prostate and prostate cancer tissues**. An anti-HS monoclonal antibody (Abcam) were used; normal prostate – epithelial prostate cancer cells and extracellular matrix are stained (brown); prostate cancer – two images from the same prostate tumor show a heterogeneity of HS expression (high and low HS expression in morphologically different epithelial prostate cancer cells are shown on the left and right images, respectively), low HS expression in extracellular matrix is shown (light brown); representative prostate cancer specimen is presented; magnification ×200.

Heparan sulfate expression in normal human prostate tissue and prostate tumors was determined using immunohistochemical staining with anti-HS mouse monoclonal antibody (Figure 1).

The obtained results show that HS expression is changed in prostate tumors compared with the normal human prostate tissue mainly due to decreased HS content in tissue stroma and heterogeneous HS expression in different cells/tissue compartments even within the same prostate cancer specimen (Figure 1).

**Figure 2:**
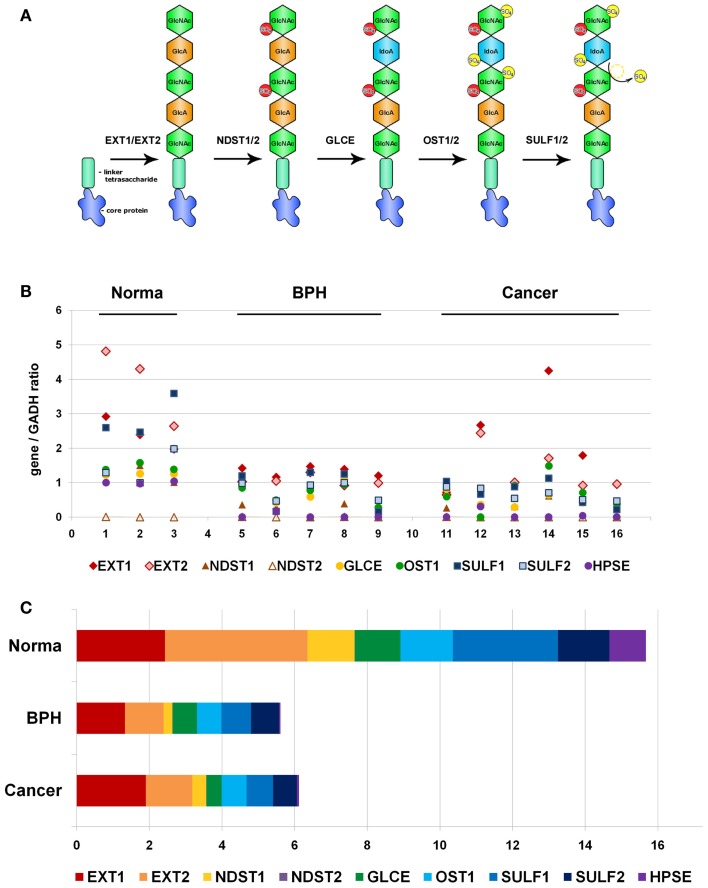
**Transcriptional patterns of HS-synthetic enzymes in normal human prostate tissue, benign prostate hyperplasia, and prostate tumors**. **(A)** Schematic organization of the HS-biosynthetic machinery. **(B)** Comparative analysis of relative transcription activity of key genes involved in HS biosynthesis, 1–16-patients. **(C)** Relative activity of HS-metabolic machinery in normal prostate tissue, BPH, and prostate tumors. Stacked column compares the contribution of each value to a total across categories. BPH, benign prostate hyperplasia.

Because of non-template-driven HS biosynthesis, HS-metabolic machinery and transcriptional disbalance of a number of HS biosynthesis/modification-involved genes could be the first potential candidate for the observed heterogeneity of HS expression in prostate tumors. To test the hypothesis, we have selected a list of nine key genes for HS metabolism, involved in both synthesis and modification/degradation of HS chains (EXT1, EXT2, NDST1, NDST2, GLCE, OST1/HS3ST1, SULF1, SULF2, HPSE) (Figure 2A).

### Heparan sulfate biosynthesis is impaired in BPH and prostate cancer tissues

RT-PCR analysis was used to study expression of all the genes in clinical specimens of normal prostate tissue, BPH, and prostate tumors. Specific primers and PCR conditions are listed in Table 1. Expression level of each gene was determined from the intensity of the amplified DNA fragments normalized to that of GAPDH and plotted onto graph (Figure 2B).

According to the RT-PCR data, the overall activity of HS-metabolic machinery both in BPH and prostate tumors was significantly lower than that in normal prostate tissue (ANOVA, Sig. 0.00007). However, specific transcriptional patterns of HS metabolism-involved genes were shown in BPH and prostate cancer tissues (Figures 2B,C). While all the genes, except NDST2, were expressed in relatively balanced ratio in normal and BPH specimens, disbalance of the transcriptional patterns in favor to HS chains elongation stage (EXT1/EXT2) was observed in prostate tumors, where total expression level of EXT1 and EXT2 expression constituted near a half of the total enzymes expression.

The obtained results clearly show that transcriptional activity of HS-metabolic system is impaired by two to three times in pathological prostate tissues, and BPH and prostate tumors possess different expression patterns of HS biosynthesis/modification-involved genes. Heterogeneity of prostate tumors on this parameter could result from an existence of cancer cell sub-types with different activity of HS-metabolic system, contributing to high intratumor heterogeneity in prostate cancer.

### Prostate cancer cells LNCaP, PC3, and DU145 have different transcriptional patterns of HS-metabolic system

**Figure 3:**
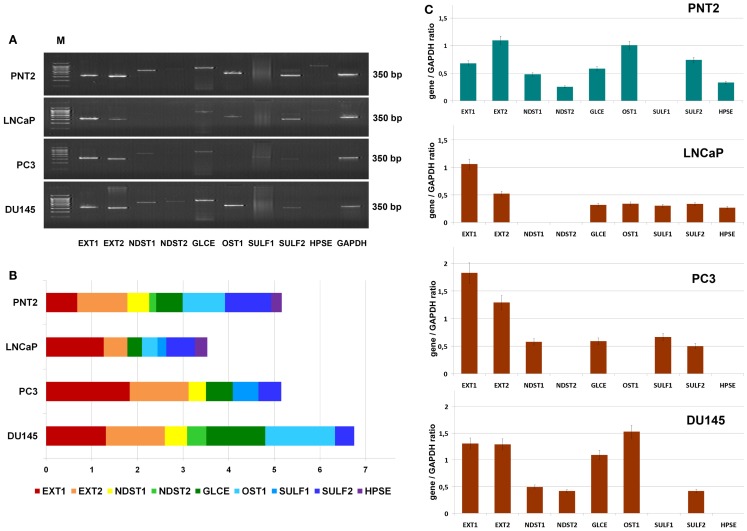
**Transcriptional patterns of HS-metabolic enzymes in different prostate cancer cell lines**. **(A)** Representative gels showing the expression levels of HS metabolism-involved genes amplified by RT-PCR. **(B)** Transcription patterns of HS-biosynthetic machinery in the cell lines. Stacked column compares the contribution of each gene to an overall expression pattern across categories. **(C)** Expressions of key HS-metabolic enzymes in normal and cancer human prostate cells. The genes expression levels normalized to that of *GAPDH*. Bars represent the mean ± SD from triplicate experiments (OriginPro 8.1).

To study a possible heterogeneity of prostate cancer cells in terms of HS metabolism activity further, transcriptional patterns of HS-metabolic enzymes were determined in morphophysiologically different prostate cancer cell lines *in vitro*. Hormone-dependent non-metastatic cell line LNCaP, hormone-independent metastatic cell lines PC3 and DU145, and normal prostate cells PNT2 were used in this experiment (Figure 3).

The expression pattern of the genes in normal prostate epithelial cells, PNT2, were most balanced, with comparable contribution of each gene into common transcriptional activity of HS-metabolic system. However, there was a significant difference in the patterns between the LNCaP, PC3, and DU145 cancer cell lines (Figures 3B,C). Overall expression levels of the genes in different cancer cells were both lower (LNCaP), similar (PC3), or higher (DU145) than in PNT2 cells, up to twofold difference between LNCaP and DU145 cells. These quantitative changes in transcriptional activity of HS-metabolic machinery were accompanied by qualitative changes in the composition and ratio of the different genes expressed in the cells. The cell lines were different in terms of relative contribution of HS chain elongation (EXT1/2) and modification (NDST1/2, GLCE, OST1, SULF1/2) stages. If “elongation-related” genes were more actively transcribed in PC3 cells, “modification-related” genes showed higher total expression level in DU145 cells (Figure 3).

In summary, the results show that LNCaP, PC3, and DU145 prostate cancer cell lines differ in the transcriptional activity and specific patterns of HS-metabolic system. However, no impairment of HS-metabolic system in prostate cancer cells was observed compared with the normal prostate PNT2 cells *in vitro*.

### HS-metabolic system is dynamically regulated by different external and internal stimuli in cell type-dependent manner

**Figure 4:**
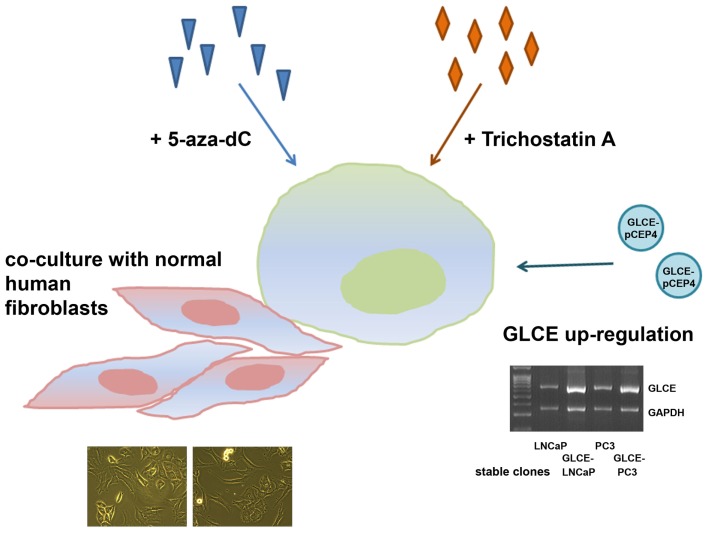
**Schematic representation for the experiment on stability/instability of the expression patterns of HS-metabolic enzymes in prostate cancer cells**. LNCaP and PC3 prostate cancer cells were used, 5-aza-dC – 5-deoxyazacytidine, multiplex RT-PCR gel showing *GLCE* expression in LNCaP and PC3 cells stably transfected with the pCEP4 or GLCE–pCEP4 plasmids (GLCE-LNCaP, GLCE–PC3) is presented.

However, to exclude the possibility that the average 30–40% differences in transcriptional activity of HS-metabolic system between the cell lines might result from physiological variations of the cells states, an ability of the system to dynamically react to different external or internal stimuli was studied. LNCaP and PC3 cells were treated with chemical drugs (5-deoxyazacytidine or Trichostatin A), underwent the internal activation of one of the system members (GLCE) or co-cultivation with another cell type (fibroblasts) (Figure 4).

**Figure 5:**
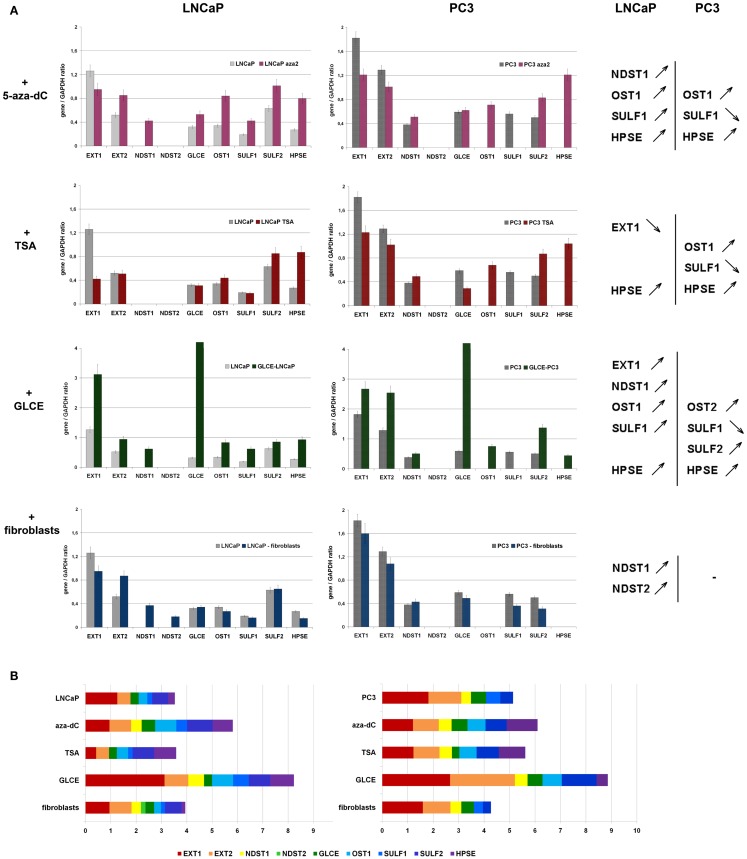
**Stability of HS-metabolic system upon different external and internal stimuli**. **(A)** Expressions of key HS metabolism-involved genes in LNCaP and PC3 prostate cancer cells before and after the treatments with 5-aza-deoxycytidine (+5-aza-dC), Trichostatin A (+TSA), ectopic over-expression of GLCE (+GLCE), co-culture with human fibroblasts (+fibroblasts). The genes expression levels normalized to that of *GAPDH*. The graphs show the mean expression levels from triplicate experiments (±s.d.) (OriginPro 8.1). Genes up-regulated or down-regulated >2-fold are shown. **(B)** Relative activity of HS-metabolic machinery before and after the treatments. Stacked column compares the contribution of each value to a total across categories.

GLCE–LNCaP and GLCE–PC3 stable clones were used for the experiment, GLCE up-regulation in the clones was confirmed by multiplex RT-PCR, the presence of GLCE protein was verified by immunocytochemical staining (23). In the co-culture experiment, prostate cancer cells and fibroblasts were cultivated together with a starting ratio 1:1 for 48–72 h. After co-cultivation, LNCaP or PC3 prostate cancer cells and fibroblasts were separated using magnetic separation system (Miltenyi Biotec). Transcriptional patterns of key HS-metabolic machinery were determined in LNCaP and PC3 prostate cancer cells by RT-PCR before and after the treatments (Figure 5). Twofold differences were taken as significant.

According to the obtained results, HS-metabolic system is able to dynamically react to different external or internal stimuli in cell type-dependent manner.

LNCaP cells were specifically affected by the treatments – TSA or fibroblasts co-cultivation did not change the levels of transcriptional activity of HS metabolism-involved genes but re-balanced it toward post-synthetic editing of HS structure (TSA) or NDST1/2 up-regulation (fibroblasts co-cultivation), while the 5-aza-dC treatment and ectopic expression of GLCE resulted in 50% and twofold overall activation of HS-metabolic system, respectively.

PC3 cells were much less sensitive to the applied stimuli and all fluctuations in the expression levels lay within the 25–30% interval. Only ectopic expression of GLCE was able to activate HS biosynthetic machinery up to 1.5–2-fold.

In general, internal up-regulation of one of the HS metabolism-involved genes (GLCE) had the most prominent effect on overall transcriptional activation of other genes in the machinery, suggesting an existence of feedback mechanism for the control of coordinated expression of HS-metabolic system as a whole.

Taken together, the results show decreased transcriptional activity and different expression patterns of HS-metabolic machinery in human BPH and prostate cancer tissues *in vivo*. Specific transcriptional patterns of HS metabolism-involved genes are shown also for different prostate cancer cell lines (LNCaP, PC3, and DU145). Androgen-dependent non-metastatic LNCaP cells and androgen-independent metastatic PC3 cells differ on their ability to dynamically react to the different external stimuli (5-deoxyazacytidine or Trichostatin A treatments, co-cultivation with fibroblasts) – LNCaP cells are sensitive to the stimuli, whereas PC3 cells are not. Only ectopic GLCE over-expression activates transcription of HS-metabolic system in both LNCaP and PC3 cell lines, suggesting internal up-regulation of one of the HS-biosynthetic machinery-involved genes as the most effective way for activation of HS-metabolic system in prostate cancer cells.

## Discussion

In this study, a complex approach to investigation of transcriptional activity of HS-metabolic system in normal and pathological prostate tissues was applied. However, the obtained results on the impairment of expression of HS-metabolic genes in BPH and prostate tumors (Figure 2) need a careful interpretation due to complex nature of prostate tissue and presence of other cell types contributing into common expression profiles of HS metabolism-related genes.

Comparative analysis of the expression data for prostate tissue samples (Figure 2) and all epithelial prostate cancer cell lines (Figure 3) shows a similar expression activity of HS-metabolic system in cancer tissues *in vivo* and prostate epithelial cancer cells *in vitro*. However, normal prostate epithelial cells, PNT2, also have a similar activity, which is threefold lower than that of normal prostate tissue *in vivo* (Figures 2,3), suggesting an important contribution by other tissue components like fibroblasts, lymphocytes, macrophages, etc to overall expression pattern of HS metabolism-related genes in normal prostate tissue *in vivo*. Possibly, stromal fibroblasts are the main source for extracellular proteoglycans in normal tissue, and an impaired expression of HS metabolism-involved genes in prostate cancer-associated fibroblasts (CAFs) significantly contributes to the overall impairment of HS-biosynthetic system in prostate tumors. Several other studies show inhibition of production of extracellular matrix (ECM) components by CAFs. For example, an expression of collagens I, II, III, IV, fibromodulin, and proteoglycans (decorin, biglycan, lumican) in stromal cells, when grown in the presence of two metastatic prostate cancer cell lines PC3 and DU145, is significantly down-regulated (35). Down-regulation of procollagens I, III, IV, fibronectin, and CS proteoglycans (5–10-fold) are detected in tumorigenic NbF-1 cells versus non-tumorigenic NbF-1 cultures (36). Close metabolic cooperation between fibroblast and epithelial cells, involving the production of growth factors by the epithelial cells and the production of extracellular matrices and growth factors by the fibroblasts, is important in promoting prostate tumor growth *in vivo* (37). The results of the present study are consistent with the data on decreased expression of the main protein components of ECM by CAFs, and extend those with parallel impairment of biosynthetic machinery for polysaccharide parts of the proteoglycans.

Interestingly, the overall inhibition of HS-metabolic system in prostate cancer is realized through the coordinated changes in the expression levels of all HS metabolism-involved genes but not at the expense of an individual one. The results fit well with GAGosome concept, where HS biosynthesis is driven by a physical complex of interacting enzymes. For example, EXT1 and EXT2 form a hetero-oligomeric complex *in vivo* that leads to the accumulation of both proteins in the Golgi apparatus (38); EXT1 and EXT2 affect the amount of NDST1 present in the cell, which, in turn, greatly influences HS structure, and there is a direct physical interaction between EXT2 and NDST1 (10); lowered expression of NDST1 results in a higher sulfate content of heparin synthesized (11); Hsepi forms a complex with Hs2st and Hs6st in S2 cells, raising the possibility that this complex formation contributes to the close functional relationships between these enzymes (39). Biosynthetic activity of the GAGosome is tightly associated with physiological properties of the cells: stromal Ext1-levels modulate tumor cell proliferation and affect the interstitial fluid pressure in a 3-D spheroid model (40); Ext1 mutant fibroblasts displayed reduced ability to attach to collagen I and to contract collagen lattices, decreased phosphorylation of ERK1/2 in response to FGF2 stimulation (whereas neither PDGF-BB nor FGF10 signaling was significantly affected), and reintroduction of Ext1 rescued HS chain length, FGF2 signaling, and the ability of the fibroblasts to contract collagen (41); EXTL2 functions to suppress GAG biosynthesis that is enhanced by a xylose kinase and the EXTL2-dependent mechanism that regulates GAG biosynthesis might be a “quality control system” for proteoglycans (42). The results of the present study stay in line with the concept of GAGosome as a functional enzymatic system, and show that the system seems to be coordinately regulated at the transcriptional level.

These results are supported by *in vitro* experiments on sensitivity of the transcriptional patterns of HS metabolism-involved genes to different external or internal stimuli. The balanced activation of HS-biosynthetic machinery in prostate cancer cells upon the ectopic over-expression of GLCE (Figure 5B) was evidently more profound than an influence of chemical drugs or presence of other cell types alongside. An independent effect for LNCaP and PC3 cell lines support an existence of a special molecular mechanism tightly controlling overall transcriptional balance of HS-metabolic machinery, possibly through its regulation as a single transcriptional group or/and with a positive/negative feedback regulation by HS structure.

The regulation seems to still work in LNCaP cell line, in spite of the decreased overall transcriptional activity of HS-metabolic system in the cells, and deregulated in more aggressive androgen-independent metastatic PC3 cells, which almost lose the ability to respond to the stimuli (Figure 5).

Possibly, the ability of HS-metabolic machinery to react dynamically to external stimuli is physiologically normal and vital, and the main problem could be insensitivity to microenvironmental influences rather than physiological fluctuations in HS-biosynthetic system activity. Developmental data are good points in favor of the hypothesis. Changes in activity of HS-biosynthetic machinery are shown during developmental process, when some enzymes are activated and other inactivated during development, creating developmentally appropriate HS structures (33, 34). These changes may be regulated via unknown yet molecular mechanism, possibly involving a feedback through the structure of cell surface and ECM proteoglycans. Additional studies are necessary in this field.

Taken together, the results of the present study strongly suggest that HS-metabolic system is involved in prostate carcinogenesis, and may be a promising target for prostate cancers diagnosis and treatment.

## Conflict of Interest Statement

The authors declare that the research was conducted in the absence of any commercial or financial relationships that could be construed as a potential conflict of interest.
